# Supplementary material for: Causal interplay between lactose intolerance and gut microbiota: a combined bidirectional Mendelian randomization and in vivo validation study
Source: Front Nutr. 2026 Jun 1;13:1803337. doi: 10.3389/fnut.2026.1803337 (PMC13265576; doi:10.3389/fnut.2026.1803337)
Supplement: Supplementary file 1 [file Data_Sheet_1.zip › supplementary materials/Forward/leave one out cross validation/ebi-a-GCST90027518.finngen_R12_E4_LACTONAS.pdf]

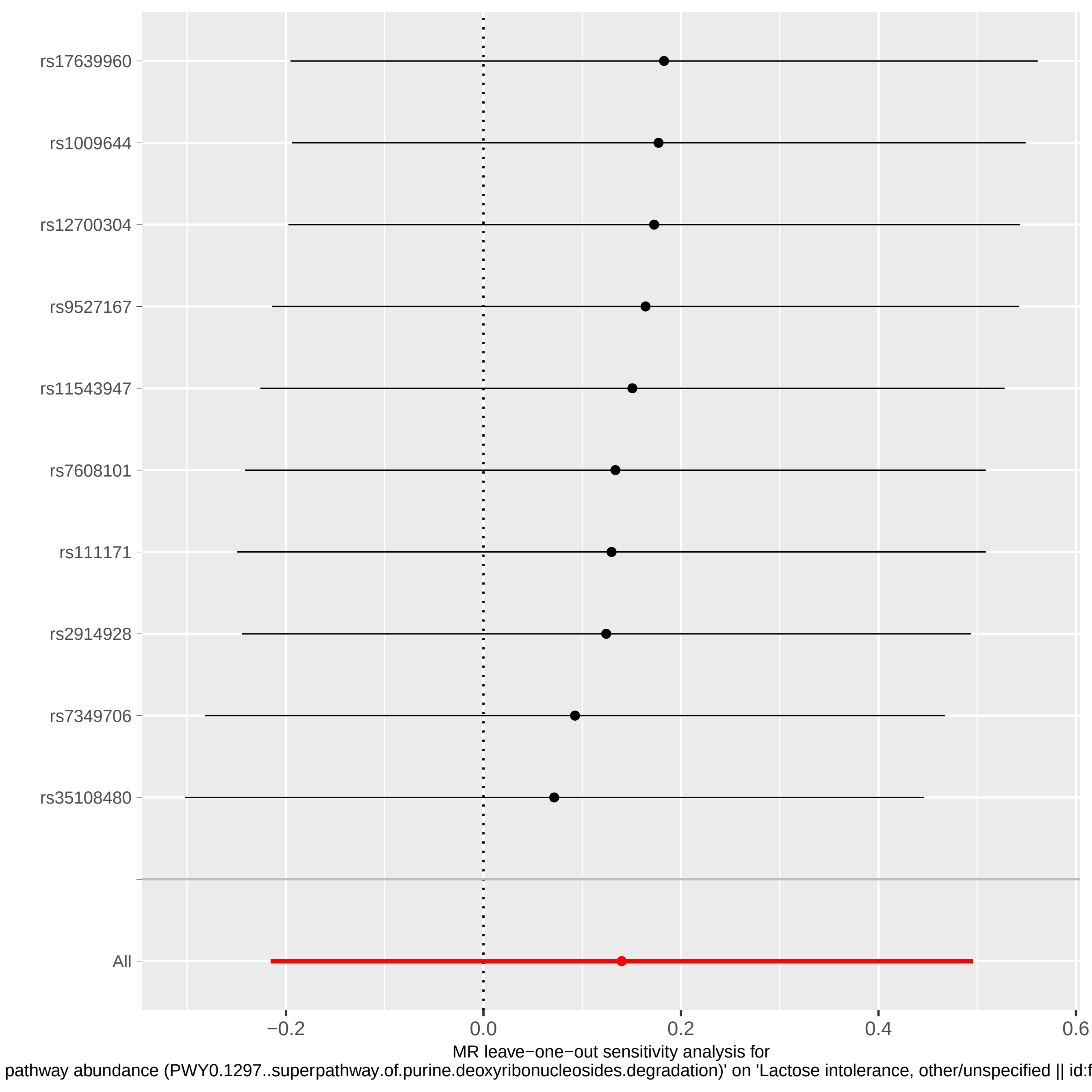
rs17639960
rs1009644
rs12700304
rs9527167
rs11543947
rs7608101
rs111171
rs2914928
rs7349706
rs35108480
All
−0.2
0.0
0.2
0.4
0.6
MR leave−one−out sensitivity analysis for
pathway abundance (PWY0.1297..superpathway.of.purine.deoxyribonucleosides.degradation)' on 'Lactose intolerance, other/unspecified || id:f
